# Sudden unexpected fatal encephalopathy in adults with *OTC* gene mutations-Clues for early diagnosis and timely treatment

**DOI:** 10.1186/s13023-014-0105-9

**Published:** 2014-07-16

**Authors:** Catia Cavicchi, Maria Alice Donati, Rossella Parini, Miriam Rigoldi, Mauro Bernardi, Francesca Orfei, Nicolò Gentiloni Silveri, Aniello Colasante, Silvia Funghini, Serena Catarzi, Elisabetta Pasquini, Giancarlo la Marca, Sean David Mooney, Renzo Guerrini, Amelia Morrone

**Affiliations:** 1 Molecular and Cell Biology Laboratory, Pediatric Neurology Unit and Laboratories, Neuroscience Department, A. Meyer Children’s Hospital, Viale Pieraccini 24, Florence, 50139, Italy; 2 Metabolic and Muscular Unit, A. Meyer Children’s Hospital, Florence, Italy; 3 Rare Metabolic Diseases Unit, Department of Pediatrics, Fondazione MBBM, San Gerardo Hospital, Monza, Italy; 4 Department of Medical and Surgical Sciences, University of Bologna, Bologna, Italy; 5 Intensive Care Unit, S. Maria della Misericordia Hospital, Perugia, Italy; 6 Department of Emergency Medicine, School of Medicine, Catholic University of the Sacred Heart, Rome, Italy; 7 Intensive Care Unit, ASL Salerno 2, Eboli Hospital, Eboli, Italy; 8 Newborn Screening Biochemistry and Pharmacology Laboratory, Pediatric Neurology Unit and Laboratories, Neuroscience Department, A. Meyer Children’s Hospital, Florence, Italy; 9 Department of Neurosciences, Psychology, Pharmacology and Child Health, University of Florence, Florence, Italy; 10 Buck Institute for Research on Aging, Novato, CA, USA; 11 Pediatric Neurology Unit and Laboratories, Neuroscience Department, A. Meyer Children’s Hospital, Florence, Italy

**Keywords:** Urea Cycle Disorders (UCD), Ornithine transcarbamylase deficiency (OTCD), Late onset OTCD, OTC gene mutations, Hyperammonemic encephalopathy, Environmental triggering factors for hyperammonemia

## Abstract

**Background:**

X-linked Ornithine Transcarbamylase deficiency (OTCD) is often unrecognized in adults, as clinical manifestations are non-specific, often episodic and unmasked by precipitants, and laboratory findings can be normal outside the acute phase. It may thus be associated with significant mortality if not promptly recognized and treated. The aim of this study was to provide clues for recognition of OTCD in adults and analyze the environmental factors that, interacting with *OTC* gene mutations, might have triggered acute clinical manifestations.

**Methods:**

We carried out a clinical, biochemical and molecular study on five unrelated adult patients (one female and four males) with late onset OTCD, who presented to the Emergency Department (ED) with initial fatal encephalopathy. The molecular study consisted of *OTC* gene sequencing in the probands and family members and *in silico* characterization of the newly detected mutations.

**Results:**

We identified two new, c.119G>T (p.Arg40Leu) and c.314G>A (p.Gly105Glu), and three known *OTC* mutations. Both new mutations were predicted to cause a structural destabilization, correlating with late onset OTCD. We also identified, among the family members, 8 heterozygous females and 2 hemizygous asymptomatic males. Patients' histories revealed potential environmental triggering factors, including steroid treatment, chemotherapy, diet changes and hormone therapy for in vitro fertilization.

**Conclusions:**

This report raises awareness of the ED medical staff in considering OTCD in the differential diagnosis of sudden neurological and behavioural disorders associated with hyperammonemia at any age and in both genders. It also widens the knowledge about combined effect of genetic and environmental factors in determining the phenotypic expression of OTCD.

## Background

Ornithine transcarbamylase deficiency (OTCD; OMIM 311250), the most common urea cycle disorder (UCD), is caused by a defect of the mitochondrial ornithine transcarbamylase (OTC, EC 2.1.3.3). The mature OTC enzyme, a trimer, catalyses the synthesis of citrulline from carbamyl phosphate (CP) and ornithine (ORN) in the liver and small intestine [[1]].

OTCD is an X-linked disorder due to deleterious mutations in the *OTC* gene in Xp21.1 and is characterized by high molecular heterogeneity with about 435 mutations having been described (HGMD: http://www.biobase-international.com/product/hgmd). Severe *OTC* gene mutations with no residual enzyme activity in hemizygous males lead to hyperammonemic coma in the neonatal period or in early infancy, which is often fatal. Some affected males may, however, exhibit a delayed onset of the disease, the so called *“late onset OTCD”* (LO-OTCD) [[2]]. The number of reported LO-OTCD patients is increasing [[3]], but only a few had remained essentially asymptomatic until acute onset in adulthood. In hemizygous male patients the phenotype is determined by several conditions, including the type of mutation but also other yet unknown factors such as environment and/or other genes. In heterozygous females phenotypic severity is also influenced by the X-inactivation pattern [[1]].

Without early diagnosis and intervention, the prognosis of OTCD with acute hyperammonemia is poor. The emergency management of hyperammonemia is based on: a) reversal of the catabolic state through glucose supplementation and halted protein intake, b) pharmacological removal of ammonia by sodium benzoate, sodium phenylacetate or sodium phenylbutyrate administration and c) extracorporeal detoxification of ammonia by dialysis [[4],[5]]. L-arginine and/or L-citrulline supplementation promote ammonia excretion through the urea cycle [[4]]. Hypothermia may be a neuroprotective measure, but its efficacy has not yet been proven in randomized controlled trials [[6]]. Guidelines for the diagnosis and management of UCD have been recently drawn up in order to provide a general consensus to guide practitioners and set standards of care [[4]].

We report a clinical, biochemical and molecular study of five adults with OTCD who developed *de novo* fatal hyperammonemic encephalopathy. We also present the *in silico* characterization of the newly detected *OTC* gene mutations and discuss the environmental events that, interacting with *OTC* mutations, might have triggered acute clinical manifestations.

## Methods

### Patients

Patients were adults referred to our diagnostic laboratory because of clinical suspicion of a UCD, and their relatives. Informed consent to the investigation, according to the Declaration of Helsinki and approved by the Human Research Ethics Committee of the Meyer’s University Hospital, was obtained from all subjects.

### Biochemical assays

Plasma amino acid analysis was performed by ion-exchange chromatography on a Biochrom 30 amino acid analyzer (Cambridge, UK) using the manufacturer’s standard protocol.

Quantification of orotic acid in urine was performed by LC–MS/MS (Toronto, Canada) as previously described [[7]].

### Detection of *OTC* gene mutations

Genomic DNA was isolated from peripheral blood using a QIAsymphony instrument as recommended by the manufacturer (Qiagen, Hilden, Germany). The entire coding region and intron-exon boundaries of the *OTC* gene were amplified by PCR as previously reported [[8]]. PCR fragments were separated on a 2% agarose gel, visualized with a UV transilluminator and then purified using Exo-SAP-IT (USB Corporation, Cleveland, OH, U.S.A).

Mutation analysis was performed by direct sequencing of the double-stranded purified products using the BigDye Terminator v1.1 Cycle Sequencing Kit (Applied Biosystems, Foster City, U.S.A.). Sequencing reactions were purified using Sephadex G-50 Fine (GE Healthcare, Little Chalfont, UK) and capillary electrophoresis was performed on ABI PRISM 3130 Genetic Analyser (Applied Biosystems) as recommended by the manufacturer.

All mutations are described according to guidelines of the Human Genomic Variation Society (HGVS) (http://www.hgvs.org/mutnomen/) and using the NM_000531.3 and NP_000522.3 reference sequences (http://www.ncbi.nlm.nih.gov/gene/).

### Screening of new OTC mutations

The Human Gene Mutation Database (HGMD) (http://www.biobase-international.com/product/hgmd) was analyzed for investigating the novelty of mutations identified. The two new point mutations were subsequently examined in the 1000 Genomes project database (http://browser.1000genomes.org/index.html). In addition, we screened by sequencing analysis the *OTC* gene of 100 DNA samples from healthy males to estimate the frequency of new mutations in the Italian population.

### Bioinformatic analyses of new *OTC* mutations

We downloaded the crystal structure for human OTC protein [PDB: 1OTH] for structural analysis of the mutant OTC enzyme. The 2D structure boundaries and solvent accessibility were calculated with the Stride program [[9]].

Stability changes in OTC protein introduced by mutations were predicted by IMutant 2.0 [[10]]. Structure-based predictions were performed *in silico* at pH 7 and 37°C.

Knowledge about catalytic residues and substrate binding sites was obtained from the literature [[11],[12]]. Effects of mutations on the trimeric protein were predicted based on PDB entry 1FVO. Inter-residue contacts were studied with the CSU program [[13]].

For conservation analysis, 18 homologous sequences (10 from eukaryotes and 8 from bacteria) were aligned by MAFFT E-INS-I [[14]]. Conservation scores were obtained from the ConSurf program (Bayesian paradigm) [[15]].

The pathogenicity and functional effects of the new *OTC* mutations were evaluated by SNAP (http://rostlab.org/services/snap/), PolyPhen-2 (http://genetics.bwh.harvard.edu/pph2/) and MutPred (http://mutpred.mutdb.org/). The probability threshold for pathogenicity was set to the default value 0.05 for all tools.

## Results

### Clinical and biochemical investigation

Four Italian men (Pts 1 to 4) and one woman (Pt 5), aged 21 to 66 years, developed initial and fatal hyperammonemic encephalopathy due to undiagnosed LO-OTCD. Their main clinical and laboratory findings are summarized in Tables 1 and 2. Detailed case reports are available in Additional file 1: Text S1.

**Table 1:** Clinical findings of adult OTCD patients with fatal encephalopathy

	**Pt 1 (M)**	**Pt 2 (M)**	**Pt 3 (M)**	**Pt 4 (M)**	**Pt 5 (F)**
**ACUTE EPISODE**					
Age at fatal acute episode	45 y	44 y	21 y	66 y	34 y
Duration of fatal episode	9 d	30 d	15 d	5 d	22 d
Duration of coma	7 d	1 d (first coma); 7 d (second coma)	12 d	2 d	20 d
**FAMILY HISTORY OF METABOLIC DISORDERS**	Negative	Negative	Negative	Negative	Negative
**PAST MEDICAL HISTORY**	Cholecystectomy; Crohn disease; Carpal tunnel syndrome; Sciatica	Chronic Hepatitis B with fluctuating hypertransaminasemia and prothrombin deficiency	Negative	Hypertension; Colon cancer (colectomy and oxaliplatin and capecitabine drugs); Hiccup after chemotherapy	Drowsiness during menstruation
**DIET PRACTISE**	Regular	Vegetarian	Diet rich in vegetables	Diet rich in vegetables	Regular
**PROBABLE TRIGGERING EVENT**	Cortisone therapy for joint pains	Diet change and poor feeding (8 kg lost in a month) after dental surgery	Diet change: a meal of fish in a Chinese restaurant	Chemotherapy (Oxaliplatin, Capecitabine)	Infertility hormone therapy and ICSI-ET, but without an ongoing pregnancy
**MISDIAGNOSIS AT ONSET**	Depression; Unspecified hepatopathy	Infectious disease; Intestinal pseudo-obstruction; Portosystemic shunts	Poisoning; Infectious diseases; Drug abuse	Poisoning; Ischemia; Cancer; Hepatitis	Narcolepsy; Cancer
**SIGN AND SYMTOMS OF ACUTE EPISODE**					
Abdominal pain/Vomiting	No/Yes	Yes/Yes	Yes/Yes	No/No	No/No
Headache/Vertigo	Yes/Yes	No/Yes	No/No	No/Yes	No/No
Fever	No	Yes	No	No	Yes
Food refusal	Yes	Yes	No	No	No
Seizures	No	Yes	No	Yes	Yes
Hepatomegaly	No	No	No	Mild	No
Consciousness disturbances	Confusion; Drowsiness; Coma (2 d after onset)	Irritability; Confusion; Coma lasting 24 hours (5 d after onset); Subsequent coma (20 d after)	Confusion; Drowsiness; Coma (3 d after onset)	Slurred speech; Confusion; Hallucinations; Drowsiness; Coma (3 d after onset)	Drowsiness; Coma (2 d after onset)

*Abbreviations: M* male, *F* female, *y* years, *d* days, *ICSI-ET* intra cytoplasmatic sperm injection - embryo transfer.

**Table 2:** Laboratory findings and therapy of adult OTCD patients with fatal encephalopathy

	**Pt 1 (M)**	**Pt 2 (M)**	**Pt 3 (M)**	**Pt 4 (M)**	**Pt 5 (F)**
**LABORATORY DATA**					
Total bilirubin (mg/dl, n.v. <1.2)	1.4	5.4	7.9	Normal	2.8
ALT; AST (U/l, n.v. < 40)	62; 55	467; 226	128; 84	127; 97	81; 95
Ammonia (first measurement - maximum value, μmol/l, n.v. 50–80)	153-411	369-845	156-377	251-1145	362-901
Respiratory alkalosis (n.v.: blood pH 7.35-7.45, pCO_2_ 35-45 mmHg, pO_2_ 75-100 mmHg)	blood pH 7.47, −, −	blood pH 7.54, pCO_2_ 30, pO_2_ 142	blood pH 7.45, pCO_2_ 32, pO_2_ 202	blood pH 7.51, pCO_2_ 27, pO_2_ 223	blood pH 7.51, pCO_2_ 26, pO_2_ 274
^P^Glutamine (μmol/l, n.v. 399–823)	2006	4580	Normal	1680	1060
^P^Citrulline (μmol/l, n.v. 17–53)	Normal	Normal	14	3	10
^P^Lysine; ^P^Proline (μmol/l, n.v. 105–236; 117–332)	702; Normal	900; 667	-	-	462; Normal
^U^Orotic acid excretion (mmol/mol creatinine, n.v. 0.2-1.1)	378	34	Normal	234	136
Neuroimaging	Normal at onset (CT); Cerebral edema (CT, 2 d later)	Normal at onset (CT); Cerebral edema (CT, 3rd d of the second coma)	Normal at onset (CT); Cerebral edema (CT, 3 d later)	Normal at onset (CT and MRI)	Normal at onset (CT); Cerebral edema (MRI, 2 d later)
**THERAPY**	PHN; Antibiotics, BCAA infusion and lactulose	PHN; BCAA infusion (first coma); Mannitol; Propofol and thiopental; UCD therapy: stop protein intake, high caloric intake, L-Arg and CVVHDF	PHN, BCAA infusion; Mannitol; Lactulose; Antibiotics; UCD therapy: SB, SPB and L-Arg	PHN; Phenytoin, thiopental and curare; UCD therapy: SB, SPB, L-Arg and CVVHDF	PHN; Midazolam, metamizole and hypothermia; BCAA infusion; UCD therapy: stop protein intake, high caloric intake, SPB, L-Arg and HD
Date of starting UCD therapy (days from onset)	None	2nd d from second coma	3rd d	3rd d	7th d
Ammonia after UCD therapy	None	Never normalized	Normal	Normal	Normal
Time for normalisation of ammonia under UCD therapy	None	None	4 d	1 d and a half	4 d

*Abbreviations: ALT* alanine aminotransferase, *AST* aspartate aminotransferase, − *not* available, ^*P*^plasma, ^*U*^urine, *CT* computed tomography, *d* days, *MRI* magnetic resonance imaging, *PHN* parenteral hydration and nutrition, *BCAA* branched chain amino acids, *UCD* urea cycle disorder, *L-Arg* L-arginine, *CVVHDF* continuous veno venous hemodiafiltration, *SB* sodium benzoate, *SPB* sodium phenylbutyrate, *HD* hemodialysis.

At the time of the acute episodes, none of the patients had a family history of metabolic disorders or had previous episodes of altered mental status. Interestingly, three patients reported preferring a low-protein diet with large amounts of vegetables.

The clinical course of acute expression of symptoms was similar in all patients, starting with gastrointestinal symptoms and/or impaired awareness, rapidly evolving into deterioration of the mental status and coma within 5 days from onset. Noticeably, in addition to hyperammonemia, hypertransaminasemia and respiratory alkalosis were observed in all patients.

The suspicion of OTCD was biochemically based on altered amino acid profiles and orotic aciduria, which is crucial for distinguishing OTCD from carbamoyl phosphate synthetase I (CPS1) and N-acetylglutamate synthase (NAGS) deficiencies.

After clinical/biochemical suspicion of UCD was established, emergency management of hyperammonemia was started in 4/5 patients, but although in 3/5 patients ammonia levels returned to normal after UCD therapy, exitus ensued.

### Molecular investigation

We confirmed OTCD in all patients by post-mortem *OTC* sequence analysis. We identified two novel mutations, c.119G > T (p.Arg40Leu) and c.314G > A (p.Gly105Glu), and three previously reported mutations, c.119G > A (p.Arg40His), c.622G > A (p.Ala208Thr) and c.829C > T (p.Arg277Trp). The results of molecular and computational analysis are summarized in Table 3.

**Table 3:** ***OTC*** **gene mutations identified in adult OTCD patients with fatal encephalopathy**

**Patient (sex; origin)**	**Mutation**	**Exon**	**Functional domain**	**Comment**	**Mutation reference**	**Genetic family testing**
**Pt 1** (M; Italy**)**	c.119G > T (p.Arg40Leu)	2	Polar/CP binding domain (S1)	CpG dinucleotide	This report	Three HT females: mother (76 y), two nieces (13 and 22 y); HM brother (50 y); WT sister
**Pt 2** (M; Italy and Japan**)**	c.119G > A (p.Arg40His)	2	Polar/CP binding domain (S1)	CpG dinucleotide; protein degradation under certain conditions; reported OTC activity 6%	[[17]]	Not performed
**Pt 3** (M; Italy**)**	c.314G > A (p.Gly105Glu)	4	Polar/CP binding domain (turn)	-	This report	HT mother (43 y); HM grandfather (80 y); WT brother
**Pt 4** (M; Italy**)**	c.622G > A (p.Ala208Thr)	6	Equatorial/ORN binding domain (H6)	CpG dinucleotide; reported OTC activity 4%	[[19]]	HT daughter (36 y)
**Pt 5** (F; Italy**)**	c.829C > T (p.Arg277Trp)	8	Equatorial/ORN binding domain (H9), flexible loop	CpG dinucleotide; increased k_m_ for ORN; reported OTC activity 5%	[[20]]	Three HT females: mother (72 y), sister (42 y) and niece (18 y); six WT females and two WT males

*Abbreviations: M* male, *F* female, *CP* carbamyl phosphate, *ORN* ornithine, *S* strand or β-sheet, *H*α-helix, *HT* heterozygous, *HM* hemizygous, *WT* wild type, *y* years of family members at the time of the proband’s acute episode.

None of the new genetic variants was present in the 1000 Genomes Project database or in the Italian control population, suggesting that they are likely to be disease-causing mutations. Noticeably, all three pathogenicity prediction tools, e.g. SNAP, PolyPhen-2 and MutPred, consistently classified both new variants as pathogenic (see Additional file 2: Table S2).

**Figure 1:**
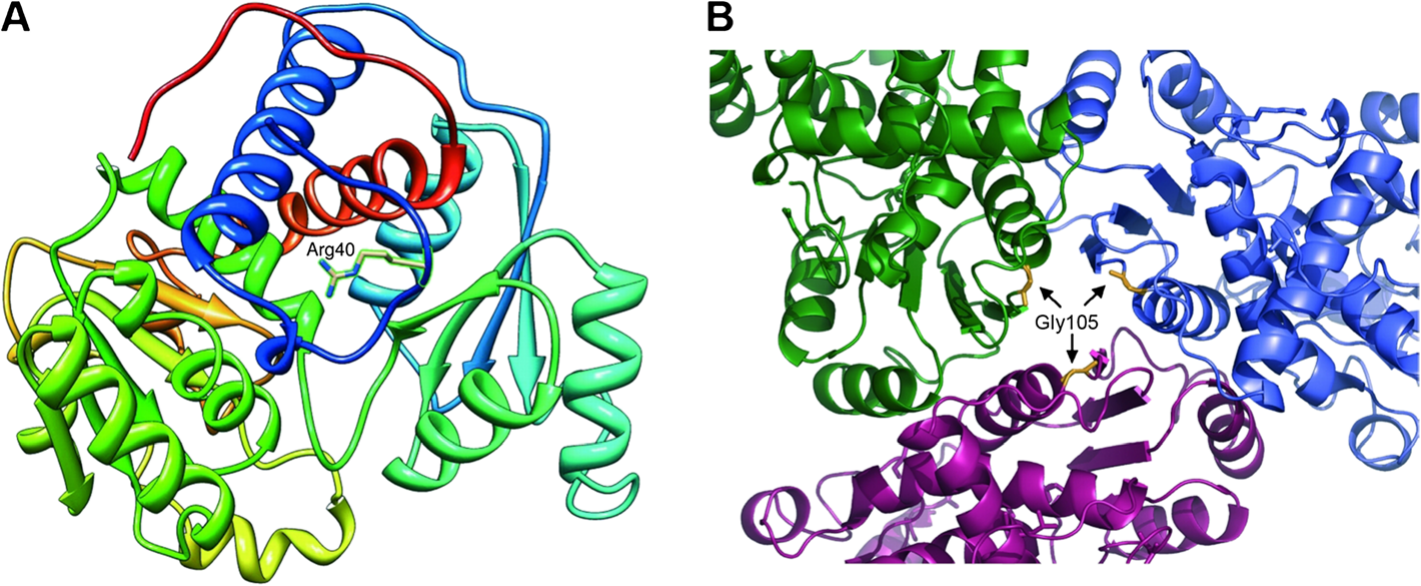
**OTC enzyme three dimensional structure mapping the new missense variants. A)** 3D visualization of the new p.Arg40Leu mutation in the OTC monomer. **B)** Trimeric assembly of OTC enzyme indicating position 105 involved in the new p.Gly105Glu mutation. Constituent monomers are colored green, blue and magenta and position 105 is colored yellow in each of them.

The novel c.119G > T (p.Arg40Leu) mutation, identified at a hemizygous level in Pt 1, affects a CpG dinucleotide in the exon which is also involved in two other known mutations, the c.118C > T (p.Arg40Cys) [[16]] and c.119G > A (p.Arg40His) [[17]]. The Arg 40 is a conserved residue of the polar/CP binding domain (residues 34–168 and 345–354) [[11]], is not in contact with the active site and its side chain is exposed to the surface (Figure 1A).

The novel c.314G > A (p.Gly105Glu) mutation was identified at the hemizygous level in Pt 3. The same nucleotide is also involved in the c.314G > T (p.Gly105Val) mutation that had previously been identified in a female with OCTD [[18]]. The Gly 105 is a conserved residue mapping in a turn of polar/CP binding domain of the OTC protein [[11]]. In the trimeric OTC enzyme the 105 position is located on the top of trimer interface and the three amino acids would be close to each other in the quaternary structure (Figure 1B).

All the known c.119G > A (p.Arg40His), c.622G > A (p.Ala208Thr) and c.829C > T (p.Arg277Trp) mutations have been previously reported in OTCD patients with residual enzymatic activity [[17],[19],[20]].

After the probands’ characterization the molecular study of the *OTC* gene was extended to family members and led to identification of 8 heterozygous females and 2 additional hemizygous asymptomatic males (Table 3).

### Retrospective analysis for potential environmental triggering factors

The retrospective analysis of the patients’ histories revealed a number of precipitating environmental factors, including therapy with steroids (Pt 1), diet changes implying poor feeding after dental surgery (Pt 2) and increased dietary protein intake plus food additives (Pt 3), chemotherapy (Pt 4) and hormone therapy for in vitro fertilization (Pt 5).

## Discussion

### Clinical and biochemical clues

OTCD is traditionally identified in pediatric patients [[21]], while late-onset presentations may remain latent for many years and often go unrecognized. As a consequence, mortality rates may be high if they are not promptly treated. An observational study of non-classical UCD including OTCD adults has been recently reported [[3]]. However, no information is available indicating the number of asymptomatic adult probands, or their clinical course or the potential role of precipitant factors. Herein we report previously asymptomatic OTCD adults to provide clues that might help recognition of OTCD in adult age, and discuss the environmental factors triggering initial and fatal hyperammonemic episodes.

The patient’s dietary preference for vegetables should be considered as an important clinical clue, since voluntary protein avoidance is a characteristic eating behavior that UCD patients adopt in order to avoid postprandial headache or drowsiness [[22]].

Special attention should be given to the biological specimen collection, since biochemical marker determination is essential for UCD diagnosis, especially during the acute episodes and also in patients with fatal outcome. In patients with acute unexplained encephalopathy is mandatory to perform blood gas analysis and plasma ammonia measurement as part of the basic work-up, even in the absence of liver dysfunction. Detection of respiratory alkalosis and hyperammonemia must address further metabolic investigations on plasma and urine to confirm or exclude UCD, without delaying the specific hyperammonemia treatment, as clinical outcome strictly correlates with the duration and peak level of hyperammonemia [[4]]. Analysis of plasma amino acids and urine orotic acid should be urgently performed in a specialist centre at initial detection of hyperammonemia. Glutamine levels and increased urinary orotic acid are the main biochemical markers for acute-phase OTCD. Citrulline and arginine levels are often low in LO-OTCD but may be normal outside the acute phase [[23],[24]].

In Pt 3 the normal values of both plasma glutamine and urinary orotic acid, detected at first measurement when only mild symptoms were present, were at odds with the suspicion of OTCD, but a final OTCD diagnosis was reached through molecular analysis. In Pt 2 we found high plasma and urinary levels of lactate during his comatose state. This finding is in line with Snodgrass 2004 [[25]] who reported lactic acidosis only in advanced phase of OTCD.

It has been reported that during acute OTCD episodes coagulopathy may occasionally appear as a consequence of liver dysfunction [[26]]. More recently, it has been emphasised that coagulation abnormalities are previously unidentified complications of OTCD also in a remission state [[27]]. In light of these findings, a diagnosis of OTCD and other UCD should be actively sought in any child or adult presenting with coagulopathy and/or liver failure of undetermined etiologies [[28]]. In Pt 2 we detected prothrombin deficiency, but is unknown if it was an expression of chronic hepatitis, or of OTCD or of both. In this patient the diagnostic process was severely complicated and delayed by many misdiagnoses, such as portosystemic shunt, as frequently described by Japanese authors [[29]].

Hyperlysinemia and hyperprolinemia, which have been reported as negative prognostic factors for OTCD [[30]], have been detected in 3 patients in our series. However, hyperprolinemia in Pt 2 may be secondary to hyperlactacidemia, probably because proline oxidase is inhibited by lactic acid [[31]].

### Molecular analysis and mutational correlations to LO-OTCD

Molecular analysis is the first choice method for confirming an OTCD diagnosis, thus it would be advantageous that the emergency department staff promptly consult a clinical biochemical geneticist or clinical geneticist, even at the initial presentation if possible (see links: http://www.orpha.net/consor/cgi-bin/index.php, https://www.eimd-registry.org/ and https://rarediseasesnetwork.epi.usf.edu/ucdc/index.htm). However, most of the *OTC* mutations are private and variants whose pathogenicity is unknown and sometimes difficult to ascertained are frequently found. Thus, *in silico* analysis, by combining different tools, may be useful for predicting variant effects.

The novel p.Arg40Leu mutation leads to destabilization of the OTC enzyme by the loss of a salt bridge or of MoRF (Molecular Recognition Feature) binding. The MoRFs represent a class of disordered regions that provide molecular recognition and binding functions to other proteins and undergo disorder-to-order transitions upon specific bindings [[32]]. The *in silico* results for p.Arg40Leu are in line with the suggestion that mutations affecting codons on the convex face of the OTC trimer, such as Arg 40, may alter the conformation of the C-terminus and hence interfere with its interaction with other proteins or the membrane [[11]].

The novel p.Gly105Glu mutation leads to a destabilization of local structures because of changes in solvent accessibility and in charge and steric hindrance, finally resulting in a perturbation of the trimer assembly. Therefore, p.Gly105Glu seems to lead to a partially functional enzyme.

The known p.Arg40His, p.Ala208Thr and p.Arg277Trp mutations are also responsible for partial OTC enzyme deficiency. Although the enzymatic assay was not performed for the novel p.Arg40Leu, *in silico* data indicating the biochemical similitude between the two mutations p.Arg40Leu and p.Arg40His and the comparable clinical presentation of patients carrying such mutations, suggest that p.Arg40Leu is also correlated with residual enzymatic activity and LO-OTCD.

Four out of five identified mutations affect CpG islands and three of them arise in arginine. Arginine has a high mutational score, likely because four of the six codons encoding it contain CpG dinucleotides, which represent mutational hot spots [[2]]. Since the novel p.Arg40Leu mutation affects the same CpG island as the known recurrent c.119G > A (p.Arg40His) mutation, it is probable that p.Arg40Leu might also occur in additional families.

### Correlation of OTCD unmasking with environmental factors

The acute disease expression in the patients we reported results from a combination of genetic and environmental factors. Exposure to new environmental or stress factors may induce an unusual nitrogen load or a significant catabolic event, which can briskly affect the patients’ homeostasis and interfere with residual OTC activity, making it no longer sufficient to remove the increased amount of waste nitrogen generated. Some medications may also cause hepatic toxicity and subsequent additional impairment of already overwhelmed OTC activity in the liver. The identification in the patients’ families of several healthy adults of both genders harboring the same mutations as the patients, corroborates the hypothesis that these mutations cause their pathogenetic consequences only when particular environmental conditions arise.

Cortisone is a glucocorticoid hormone with many therapeutic uses. High circulating concentrations of glucocorticoids are known to have a general catabolic effect by primarily enhancing protein turnover [[33]]. There are at least four reports of OTCD adults who developed acute hyperammonemic coma following steroid administration [[5],[34]–[36]].

Fasting and surgery are commonly considered dangerous conditions for OTCD patients since they cause metabolic stress and are often accompanied by reduced energy production and increased turnover of endogenous proteins [[5],[37]–[39]]. Moreover, a role of high protein diets (e.g. Atkins’ diet) in unmasking adult-onset OTCD has been previously described [[40]].

Besides a high protein intake as an obvious triggering factor in Pt 3, the possible precipitating role of monosodium glutamate (MSG) consumption, which is especially used in Asian cuisine as a flavor enhancer, could also be considered. MSG has been reported to accelerate gastric emptying of a protein-rich meal in humans, stimulating secretions from the exocrine and endocrine systems and leading to increased plasma glutamine concentrations [[41]]. Therefore, MSG ingested by our patient could have contributed to a more rapid overload of nitrogen and glutamine.

Chemotherapics are among the medications causing hepatic toxicity and affecting protein catabolism. Their side effects on the oro-gastrointestinal tract are indeed responsible for impairing dietary protein absorption and consequently accelerating degradation of endogenous proteins. A postchemotherapy hyperammonemic encephalopathy emulating OTCD has been described as a distinct syndrome of hyperammonemia in two young women with hepatocellular carcinoma, who resulted mutation-negative to *OTC* [[42],[43]]. Since insertion/deletions or deep intronic mutations were not excluded, OTCD unmasked by chemotherapy could still be possible in these patients.

The precipitating environmental factor for the female patient is not obvious, but a potential role of sex hormones or hormonal drugs seems to be very likely. Menses are considered precipitants for acute hyperammonemia in UCD [[44]] and a relationship between sex hormones and ureagenesis in a woman with UCD has been reported [[45]]. At least three hypotheses might explain the OTCD unmasking in our patient: a) the physiological changes of the endometrium during the menstrual cycle, which produce large amounts of waste nitrogen and which may explain the drowsiness occurring in conjunction with menses; b) the direct action of endogenous sex hormones and/or hormonal drugs on the urea cycle by modulation of enzymatic expression and function; c) the toxic effects of infertility medication resulting in worsening of hepatic function, since these drugs are primarily metabolized in the liver.

An additional precipitant for the metabolic decompensation in our patients might have been parenteral nutrition, which provides more proteins than the patient usually expends entirely [[46]].

## Conclusions

The diagnosis of OTCD in adults may be difficult, as clinical manifestations are non-specific and often episodic and laboratory findings can be normal outside the acute phase. Since LO-OTCD can be hidden and suddenly become symptomatic under specific environmental factors, early suspicion/diagnosis is crucial to providing effective treatment in probands and to helping prevent clinical outbursts in other family members. This report raises awareness of emergency department staff in considering OTCD in the differential diagnosis of sudden neurological and behavioural disorders associated with hyperammonemia at any age and in both genders. Molecular analysis is the method of choice for confirming an OTCD diagnosis, for uncovering the carrier status in at risk family members and for providing genetic counselling and prenatal diagnosis. Future efforts should be aimed at further widening the knowledge about combined effect of genetic factors (i.e. mutations in the OTC gene, variants in modifier genes or epigenetic features) and environmental conditions in determining the phenotypic expression of OTCD.

## Availability of supporting data

The data sets supporting the results of this article are included within the article and its additional files.

## Abbreviations

OTCD: X-linked ornithine transcarbamylase deficiency

UCD: Urea cycle disorder

CP: Carbamyl phosphate

ORN: Ornithine

LO-OTCD: Late onset OTCD

LC-MS/MS: Liquid chromatography-tandem mass spectrometry

Pt: Patient

CPS1: Carbamoyl phosphate synthetase I

NAGS: N-acetylglutamate synthase

MoRF: Molecular recognition feature

MSG: Monosodium glutamate

## Competing interests

The authors declare that they have no competing interests.

## Authors’ contributions

CC participated in the conception and design of the study, performed the molecular genetic studies, contributed to the analysis and interpretation of data and drafted the manuscript. MAD participated in the conception and design of the study, contributed to the analysis and interpretation of clinical and biochemical data and revised the article critically for important intellectual content. RP, MR, MB, FO, NGS and AC performed the acquisition of clinical findings and contributed to the discussion of data. SF and GLM performed the biochemical assays. SC contributed to the analysis and interpretation of genetic data. EP contributed to the analysis and interpretation of clinical and biochemical data and revised the article critically for important intellectual content. SDM performed the *in silico* analysis of new mutations and contributed to the discussion of genetic findings. RG contributed to the discussion of clinical data and revised the article critically for important intellectual content. AM participated in the conception and design of the study, coordinated the study, contributed to the analysis and interpretation of data and revised it critically for important intellectual content. All authors read and approved the final manuscript.

## Additional files

## Supplementary Material

Additional file 1: Text S1 Case reports. Click here for file

Additional file 2: Table S2 Bioinformatic results for the novel p.Arg40Leu and p.Gly105Glu mutations. Click here for file
