# Supplementary material for: Sudden unexpected fatal encephalopathy in adults with OTC gene mutations-Clues for early diagnosis and timely treatment
Source: Orphanet J Rare Dis. 2014 Jul 16;9:105. doi: 10.1186/s13023-014-0105-9 (PMC4304088; doi:10.1186/s13023-014-0105-9)
Supplement: Additional file 1: Text S1 — Case reports. [file s13023-014-0105-9-S1.doc]

**Additional file 1: Text S1. Case reports**

**Patient 1**

A 45-year old man was admitted to the Emergency Department because of food refusal, vomiting, vertigo, headache and cognitive slowing. Until then he had led a relatively normal life without dietary restriction. The patient had received cortisone therapy because of joint pain a few days before symptom onset. Initial laboratory work-up revealed leukocytosis. Brain computed tomography (CT) imaging and toxicology tests were negative. Acute depression was diagnosed. Within a few hours he progressively experienced negative mood and drowsiness. The following day he stopped eating and received parenteral hydration and nutrition as well as antibiotics. Blood analysis revealed hyperbilirubinemia, mild hypertransaminasemia and hyperammonemia (153 μmol/l, n.v. 50-80). A misdiagnosis of liver disease of uncertain etiology was made, thus antibiotics and hepatotoxic treatment, including BCAA and lactulose, were administered. On the third day the patient’s neurological status progressed to coma, with diffuse brain edema on CT. He was admitted to the Intensive Care Unit (ICU). His ammonia levels rose to 411 μmol/l and his blood pH was 7.47 (n.v. 7.35-7.45). His plasma amino acid profile showed increased glutamine, normal citrulline and arginine. Urine organic analysis by gas chromatography/mass spectrometry (GC/MS), revealed slightly increased levels of 4-hydroxy-phenyllactic and 4-hydroxy-phenylpyruvic acids (4 and 14 mmol/mol creatinine, respectively, n.v. <2) due to the liver failure. Urinary orotic acid was markedly increased. The patient died 9 days after hospital admission.

**Patient 2**

A 44-year old Italian-Japanese male was admitted to the Gastroenterology Unit because of dyspepsia, poor feeding, vomiting and irritability. He reported switching from a usual vegetarian diet to dairy products with poor feeding and marked weight loss (8 Kg) over the last month after receiving dental surgery. Over the next few days, he became increasingly confused and drowsy developing a comatose state with hyperammonemia (369 μmol/l) lasting one day. Laboratory work-up ruled out infectious diseases. Brain CT scan was normal. He received parenteral hydration, glucose and BCAA intravenous infusion with improvement in his neurological status. Ultrasound and CT of the abdomen showed a normal-sized liver with granular echotexture, but no focal lesions. The patient was intermittently agitated and disoriented, until dramatically plunged into coma. Blood chemistries demonstrated hyperammonemia (280 μmol/l), hypertransaminasemia, hyperbilirubinemia, hyperlactacidemia (92 mg/dl, n.v. 9-18), respiratory alkalosis, and elevated levels of creatinine, creatine phosphokinase, troponin T, aPTT-ratio and PT-INR. ECG revealed sinus tachycardia and signs of myocardial ischemia, thus metoprolol tartrate and nitroglycerin were started. Mannitol was administered due to suspicion of hyperammonemic cerebral edema. Plasma amino acid analysis showed a generalized increase in concentration of multiple amino acids, including glutamine and urinary analysis revealed increased orotic acid. These findings were consistent with OTCD and the patient started the specific UCD therapy. In the following days he experienced myoclonic seizures that were treated with propofol and thiopental. The CT scan showed diffuse cerebral edema. Despite UCD therapy, his blood ammonia was 542 μmol/l with elevated urinary lactic acid (1592 mmol/mol creat, n.v. < 25). The patient died 7 days after admission to the ICU.

**Patient 3**

A 21-year old man, with no previous relevant history, started complaining of abdominal pain and vomiting after an oriental meal of fresh fish and pasta. At admission he exhibited skin and scleral jaundice and was disoriented. Laboratory findings showed hyperammonemia (156 μmol/l), hypertransaminasemia, hyperbilirubinemia and respiratory alkalosis, but excluded infectious diseases, poisoning and drug abuse. Plasma amino acid profile showed a reduction of citrulline, normal levels of arginine, ornithine and glutamine. Also urinary orotic acid was in the normal range. Brain CT scan was normal at onset. He was put on BCAA intravenous infusion. In the following few hours his symptoms worsened and he began to feel drowsy. Two days afterwards coma occurred and blood ammonia levels rose to 377 μmol/l. He was mechanically ventilated and received mannitol, lactulose and vasopressor support. Liver ultrasound revealed normal size and increased echogenicity, without focal lesions. Brain CT scan showed cytotoxic edema with reduced ventricular size and lowered basal ganglia density. OTCD was suspected and sodium benzoate by nasogastric tube and intravenous L-arginine were started. The patient’s ammonia blood level decreased to 63 μmol/l, but after 24 hours severe hypotension, sinus tachycardia and worsening of renal function and coagulation tests were ascertained. Liver biopsy demonstrated hepatic nuclear glycogenosis associated with centrilobular sinusoidal dilatation and atrophy of the hepatic plates. Although ammonia levels normalized, the patient never regained consciousness and died 11 days later.

**Patient 4**

A 66-year old man came to the Emergency Department because of slurred speech, confusion, vertigo, hallucinations, tremors and drowsiness. He was receiving chemotherapy (oxaliplatin and capecitabine) after an operation for colon adenocarcinoma. His relatives reported that he had a dietary preference for vegetables, regular afternoon naps and frequent hiccups after colectomy, which worsened during chemotherapy. He had received chemotherapy 5 days before the acute episode. At onset mild hepatomegaly was present but was not associated with altered liver function tests. Brain scans also demonstrated no abnormalities. On the second day he developed seizures and blood chemistries demonstrated hyperammonemia (251 μmol/l) and hypertransaminasemia. His neurological status rapidly worsened to coma on the third day, thus he was moved to the ICU, intubated and mechanically ventilated, receiving continuous sedative infusions and vasopressor support. Respiratory alkalosis has been observed and ammonia levels dramatically rose to 881 μmol/l. Serological testing excluded acute viral hepatitis. Hemodiafiltration was begun to reduce ammonia but remained over 585 μmol/l. Despite treatment with phenytoin and thiopental, epileptic seizures persisted and required curarization. On the fourth day the patient became hemodynamically unstable and febrile and ammonia was 1145 μmol/l. Plasma amino acid analysis showed increased glutamine and decreased citrulline. Urinary orotic acid was markedly elevated. The patient was started on specific UCD therapy. The ammonia levels dropped into the normal range, but the patient was in terminal condition and died the following day.

**Patient 5**

A 34-year old Italian woman was admitted to the ICU after being sleepy for two consecutive days and suffering severe dehydration and undernourishment. Some months before the acute episode she received hormone therapy for *in vitro* fertilization. Upon questioning, her relatives reported regular interruption of her work during menstruations because of drowsiness and worsening of drowsiness after each hormonal stimulation. Four days prior to admission she also underwent ICSI-ET (Intra Cytoplasmatic Sperm Injection - Embryo Transfer), becoming drowsy and then unconscious. She was intubated, ventilated and received parenteral hydration and nutrition. Two days later she became febrile and suffered epileptic seizures. She was treated with midazolam, metamizole and hypothermia. On the fourth day of hospitalization BCAA was infused. Brain CT was normal at onset. Magnetic resonance, performed 2 days later, showed mild hyperdensity in the subcortical**-**frontal regions and diffuse edema. Electroencephalography (EEG) showed generalized slowing with triphasic delta waves. Repeat ultrasound and blood human chorionic gonadotropin (hCG) tests (<2.2 U/l, normal values <5) ruled out a pregnancy. The laboratory workup revealed an initial plasma ammonia level of 362 μmol/l, respiratory alkalosis, hyperbilirubinemia and hypertransaminasemia. On the seventh day ammonia rose to 901 μmol/l. Her plasma amino acid profile showed increased glutamine, lysine and methionine, decreased citrulline and normal arginine levels. Moreover, increased urinary orotic acid provided strong evidence of OTCD and the patient was started on specific UCD therapy. Although her ammonia level gradually dropped to normal, the patient never regained consciousness and died 22 days after admission.
