# Supplementary material for: Sudden unexpected fatal encephalopathy in adults with OTC gene mutations-Clues for early diagnosis and timely treatment
Source: Orphanet J Rare Dis. 2014 Jul 16;9:105. doi: 10.1186/s13023-014-0105-9 (PMC4304088; doi:10.1186/s13023-014-0105-9)
Supplement: Additional file 2: Table S2 — Bioinformatic results for the novel p.Arg40Leu and p.Gly105Glu mutations. [file s13023-014-0105-9-S2.doc]

**Additional file 2: Table S2. Bioinformatic results for the novel p.Arg40Leu and p.Gly105Glu mutations.**

| **Mutation** | **2D** | **SAS** | **CSU / function** | **Stability** | **Conservation analysis**  **(score)** | **SNAP (score^a^)** | **PolyPhen-2**  **(score^a^)** | **MutPred score^a^** | **MutPred hypothesis^b^** |
| --- | --- | --- | --- | --- | --- | --- | --- | --- | --- |
| **p.Arg40Leu** | Coil | 28% | Loss of salt bridge with Glu52 and thereby structure destabilization | Decreased | Conserved residue  (-0,974) | Non-neutral (0.87) | Possibly damaging variant (0.693); replacement of a conserved and basic residue with a hydrophobic one | 0.93 | Loss of MoRF binding  (P = 0.0433) |
| **p.Gly105Glu** | Turn | 29% | Glu likely causes trimer assembly perturbation | Increased | Conserved residue  (-1,063) | Non-neutral (0.93) | Probably damaging variant (1.000); closest contact with other chains; replacement of a small and conserved residue with a large negatively charged one | 0.84 | Gain of solvent accessibility  (P = 0.0123) |

*^a^ Amino acids with probability >0.50 are predicted to be non-neutral/disease-associated. ^b^ p is the P-value that certain structural and functional properties are impacted and scores > 0.75 and p < 0.05 are referred to as confident hypotheses. Abbreviations: SAS = solvent accessible surface; CSU = contacts of structural units; MoRF = molecular recognition feature.*
